# A Prospective Observational Study on the Clinical Outcomes of Diabetic Foot Ulcers Based on Diabetic Ulcer Severity Score at a Tertiary Care Center in Jharkhand, India

**DOI:** 10.7759/cureus.89523

**Published:** 2025-08-06

**Authors:** Krishna Murari, Khushboo Rani, Zenith Kerketta, Amit Nishant, Anish Baxla, Ujala Murmu, Neyaz Ahmad, Shreya, Ankita Mukul

**Affiliations:** 1 General Surgery, Rajendra Institute of Medical Sciences, Ranchi, IND; 2 Surgery, Rajendra Institute of Medical Sciences, Ranchi, IND; 3 Community Medicine, Rajendra Institute of Medical Sciences, Ranchi, IND

**Keywords:** amputation, diabetic foot ulcer, diabetic ulcer severity score, duss, hospitalization, prognosis

## Abstract

Background

People with long-standing or poorly controlled diabetes often experience problems in their feet due to nerve damage and poor blood circulation. These issues can make the feet more prone to injury and infection. When wounds do occur, they may not heal properly, sometimes resulting in ulcers, which may even lead to amputations of the foot if not managed properly and timely. This is a serious complication of diabetes. In order to manage the ulcers efficiently, a scoring system can be used to assess the severity and guide the treatment modality accordingly.

Methods

This was a prospective, observational study, conducted at the Department of General Surgery of the Rajendra Institute of Medical Sciences (RIMS), Ranchi, over a period of six months. A total of 150 patients who were diagnosed with diabetic foot ulcers (DFUs) were recruited for the study using a consecutive sampling method. Data collection was done using a pre-tested questionnaire to collect demographic information, medical history, and ulcer characteristics. A clinical examination was done, and the Diabetic Ulcer Severity Score (DUSS) scoring system was used to assess the severity of ulcers based on pedal pulse, bone exposure, ulcer location, and number of ulcers. Data were analysed using IBM SPSS Statistics software (Version 26, IBM Corp., Armonk, NY) with descriptive statistics and chi-square tests. A p-value of less than 0.05 was considered statistically significant. Ethical approval was taken from the Institutional Ethics Committee of RIMS.

Results

The study included 78 male (52.0%) and 72 female patients (48.0%), with most participants (39.3%) falling in the 46-60 years age group. A significant proportion (57.3%) hailed from rural areas, and 46% had a diabetes duration exceeding 10 years. Ulcer characteristics revealed that 48% were deep, 51.4% had mild infection, and 42% exhibited mild ischemia.* *The DUSS score analysis revealed that 36% of patients had a score of two, while 33.3% had a score of one. Amputation was the most common treatment outcome, seen in 59% of cases, followed by surgical debridement in 36%. There was a statistically significant association between higher DUSS scores and both longer hospital stays (χ²=64.9, p<0.00001) and a greater likelihood of amputation (χ²=36.9, p<0.00001). Importantly, all patients with DUSS scores of three or four required amputation.

Conclusion

The findings unequivocally demonstrate that higher DUSS scores are strongly associated with adverse clinical outcomes, specifically prolonged hospital stays and increased rates of lower limb amputation. The DUSS proves to be a good prognostic tool for early risk stratification, guiding timely and appropriate therapeutic interventions, but there is a need for clinical judgement and other imaging modalities for better patient care alongside the tool. The study underscores the need for early identification and aggressive management of DFUs, particularly in high-risk populations, and at lower levels of healthcare to improve patient outcomes and alleviate the burden on higher centers.

## Introduction

Diabetes mellitus (DM) is a long-term and common condition caused by problems with insulin production, insulin action, or both, leading to high blood sugar levels. It is a major global health issue affecting millions of people. As per the International Diabetes Federation (IDF), approximately 537 million adults suffered from diabetes in 2021, and the number is expected to increase to 643 million by 2030 and 783 million by 2045 [1]. The ever-increasing prevalence of diabetes in India is particularly disturbing. This has led the country to be recognized as the "diabetes capital of the world," with a predictable 17% of total global cases originating from India [2]. 

Diabetic foot ulcers (DFUs) are one of the gravest complications of diabetes. They lead to high morbidity and mortality and greatly diminish the quality of life for those affected. A DFU is a deep wound below the ankle in a person with diabetes, without regard to time of presentation. These ulcers often result from poor blood sugar control, nerve damage, poor circulation, or improper foot care. DFUs can lead to serious infections like foot bone infections (osteomyelitis) and are a major reason for lower limb amputations, making up about 50% to 70% of such cases. In India, DFUs affect around 4% to 10% of people with diabetes, and up to 60% of these ulcers can come back even after healing [3].

The development of DFUs is complex and involves several factors working together. Nerve damage (neuropathy) reduces feeling in the feet, causes foot deformities, and creates pressure points, all of which can lead to skin damage. The risk of ulcers is further increased because of the poor vascular supply and weak immune system in diabetes patients. Vascular compromise impairs tissue perfusion and hinders the wound healing process. The compromised immune system predisposes the patients to infections, which further worsens the ulcer’s prognosis. These factors are intertwined and they collectively contribute to the chronicity of ulcers in these patients and delay the healing, thereby increasing risk of severe complications, including loss of limb [4-6].

The management of DFUs often requires a multidisciplinary approach. It requires rigorous blood sugar controls, aggressive wound debridement, advanced dressings and others like bed rest, crutch-assisted gait or use of total contact casts. Some cases need surgical intervention to promote healing of chronic ulcers and to prevent recurrences. Advanced treatment modalities like hyperbaric oxygen therapy (HBOT), electrical stimulation, negative pressure wound therapy (NPWT), artificial skin, and growth factors can aid in the speedy recovery of wound ulcers. Apart from these clinical management methods, a very important aspect of prevention of DFUs and reduction of risk of complications like infections, gangrene and amputation is patient education. It is crucial for them to know the cause, effect and management in order to take care of their wounds [7,8]. 

It becomes important to assess the severity of DFUs accurately based on objective parameters and timely to predict the outcome given the emotional, physical and financial distress associated with it to the patients. The Diabetic Ulcer Severity Score (DUSS) was created by Beckert and colleagues in 2006. It is a simple clinical tool to assess a DFU severity and predict its prognosis [9]. The score evaluates important clinical parameters like ulcer depth, infection, and presence of ischemia, thereby enabling the doctor to make a clinical decision. By assessing the DUSS score when the patient presents, clinicians can approximate the likelihood of ulcer healing, potential need for surgical intervention, and the risk of amputation. It is a valuable adjunctive tool, particularly in resource-limited settings such as those found in many places in India [9,10].

The rationale for this study lies in the fact that India being the “diabetes capital” has a heavy burden of DFUs and the rush is even more in tertiary care settings with patients presenting with advanced complications, since it is not being managed at primary or secondary levels. Thus, having a reliable and standard DUSS scoring tool is imperative for better patient care. Therefore, the study aimed to evaluate the association between DUSS and clinical outcomes in patients with diabetic foot ulcers, including surgical debridement, skin grafting, and amputation. The objective of the study was to describe the demographic and clinical profiles of patients presenting with diabetic foot ulcers and to identify the risk factors that lead to delay in seeking treatment or advice for their condition.

## Materials and methods

This study was conducted from August 18, 2024 to February 15, 2025 (six months) within the Department of General Surgery of the Rajendra Institute of Medical Sciences (RIMS), Ranchi. The study population was patients with DFU presenting to the outpatient department (OPD) or emergency department at the Surgery Department in RIMS, Ranchi. The sample size was calculated based on earlier studies, which showed that about 10% of people with diabetes in India develop foot ulcers [11]. Using the formula $N = \frac{z^2pq}{d^2}$, where *Z* (for 95% confidence level) is 1.96, *p* (expected prevalence) is 10%, *q* (100-*p*) is 90%, and *d* (margin of error) is 5%, the required sample size was found to be 144. To allow for possible non-responses and to ensure reliable results, the final sample size was rounded up to 150. A consecutive sampling method was used, meaning all eligible patients who visited the surgery outpatient department (OPD) or emergency during the study period were included until 150 participants were enrolled. Data collection was started only after clearance from the Institutional Ethics Committee (IEC) of RIMS, Ranchi vide Memo No. 283/IEC, RIMS dated August 16, 2024. Strict eligibility criteria were applied to ensure the homogeneity and relevance of the study population. The confidentiality of all participants was rigorously maintained throughout the study by anonymizing their data. No invasive procedures were performed solely for the purpose of this research; all clinical interventions were part of standard patient care.

Inclusion criteria for the study were patients with a confirmed diagnosis of diabetes mellitus who presented with an ulcer in the foot, patients aged 18 years or above, of any gender, who were receiving treatment at RIMS, Ranchi, and patients whose ulcers were located specifically below the ankle. The exclusion criteria were patients suffering from critical systemic illnesses that would impede their participation or render data collection impractical, patients with coexisting venous or traumatic ulcers, pregnant women and minors due to specific ethical considerations and physiological differences. 

**Figure 1:**
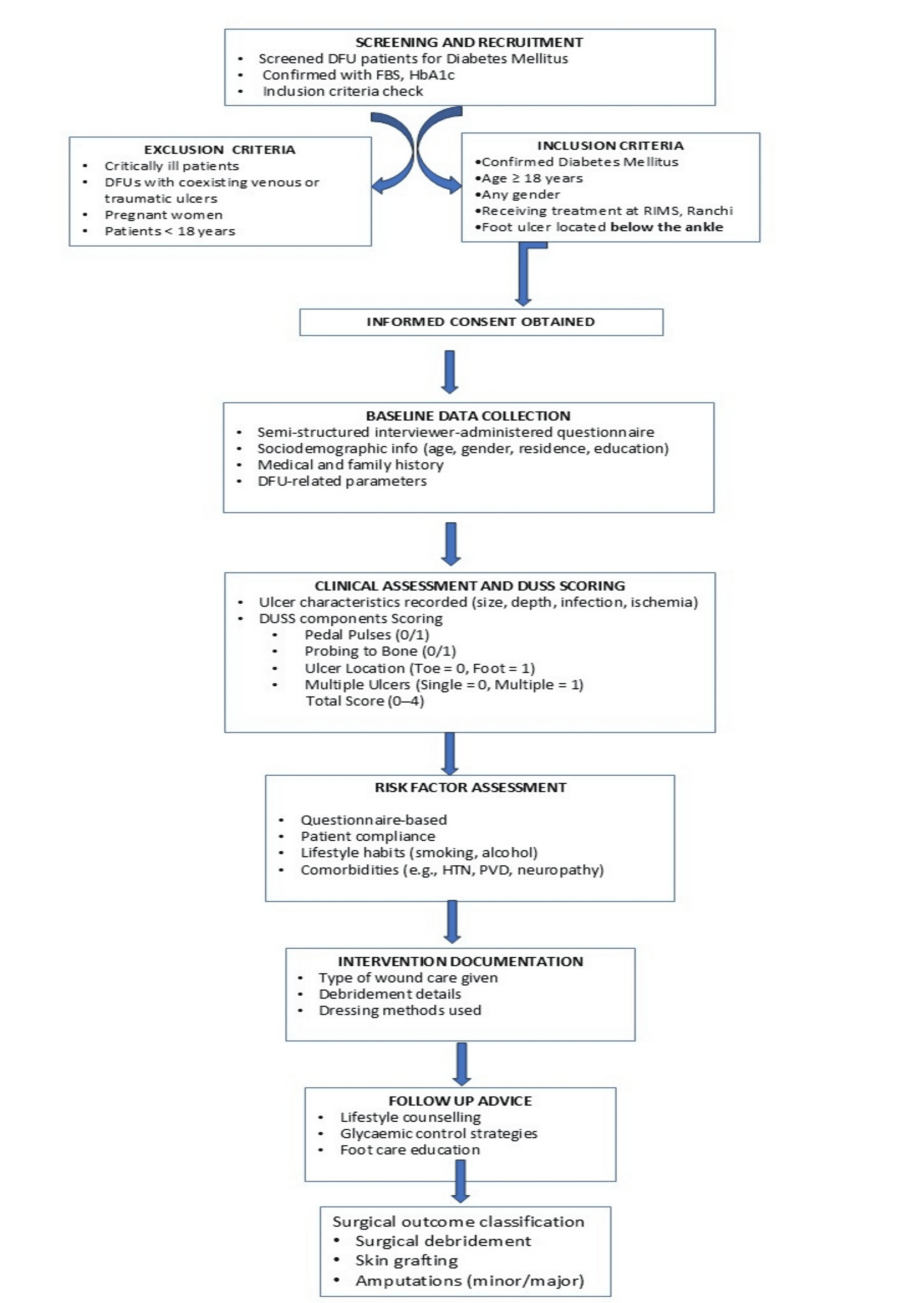
Flowchart depicting the sequential steps of data collection in the study FBS: Fasting blood sugar; HbA1c: glycated haemoglobin; DFU: diabetic foot ulcer ; DUSS: Diabetic Ulcer Severity Score; HTN: hypertension; PVD: peripheral vascular disease

The process of data collection was systematic and comprehensive, involving several stages (Figure 1).

Patients presenting with foot ulcers were initially screened for diabetes mellitus using routine laboratory investigations, including fasting blood sugar and glycated haemoglobin (HbA1c) levels. Patients who met the inclusion criteria were given full details about the study, and their informed consent was taken before they were enrolled. For baseline data, a pre-designed, semi-structured, and pre-tested interviewer-administered questionnaire was used to collect socio-demographic information such as age, gender, place of residence, and education level. The next section of the questionnaire included details about their medical and family history and other important parameters related to the DFUs and complications. A detailed clinical examination was done carefully documenting ulcer characteristics such as size, depth, presence of infection or ischemia. For scoring of DFUs, four critical clinical parameters used in the DUSS scoring tool were assessed as shown (Table 1).

**Table 1:** Diabetic Ulcer Severity Score (DUSS) scoring tool parameters.

DUSS components	Score 0	Score 1
Palpable pedal pulses	Present	Absent
Probing to bone	Absent	Present
Ulcer location	Toe	Foot (other than toe)
Number of ulcers	Single ulcer	Multiple Ulcers

The total score ranges from 0 to 4, with higher scores indicating more severe ulcers and poorer prognosis. Additional ulcer-related parameters such as depth, infection severity, and ischemia, were documented and analysed descriptively but were not included in the DUSS score calculation. Data on various potential risk factors for DFUs were gathered through the questionnaire, encompassing aspects such as patient compliance with treatment, lifestyle habits (e.g., smoking, alcohol consumption), and the presence of comorbidities (e.g., hypertension, peripheral neuropathy, peripheral vascular disease). All details of wound management provided to the patients were documented including type of debridement performed, the dressing types used, and any other measures undertaken. Participants received comprehensive counselling on lifestyle modification, strategies for optimal glycaemic control, and essential foot care practices to prevent future complications. For the purpose of analysis, surgical outcomes were classified into three categories based on the wound care given to the patients: (i) Surgical debridement (local excision of necrotic or infected tissue without removal of bone or amputation), (ii) skin grafting (use of split-thickness or full-thickness skin grafts for wound coverage following adequate ulcer bed preparation), and (iii) amputations. This was further subclassified into minor amputation (removal of toes, partial ray resections, or trans metatarsal amputations) and major amputation, which included below-knee (trans-tibial) or above-knee amputations involving loss of the limb beyond the mid-foot.

Statistical analysis

The data obtained from questionnaires and clinical evaluations were initially organized and cleaned using Microsoft Excel (Microsoft, Redmond, WA). Statistical analysis was then conducted using IBM SPSS Statistics for Windows, version 26 (IBM Corp., Armonk, NY, USA, 2019). Descriptive statistics, including frequencies and percentages, were used to summarize categorical variables such as gender distribution and DUSS score categories. Inferential statistics, particularly chi-square tests, were applied to explore associations between categorical variables. A p-value of less than 0.05 was considered statistically significant for all analyses.

## Results

The study enrolled 150 participants with DFU, and the findings are presented across various demographic, clinical, and outcome parameters.

Baseline characteristics and medical history of participants

The demographic profile of the study participants revealed an almost equal distribution between genders, with 77 men (51.3%) and 73 women (48.7%). The age distribution indicated that the largest proportion of patients, 59 individuals (39.3%), belonged to the 46-60 years age group. A significant majority of the participants, 86 individuals (57.3%), resided in rural areas, while 64 (42.7%) were from urban settings. In terms of educational attainment, 46 participants (30.7%) had a secondary education, 45 (30%) had a primary education, 32 (21.3%) were graduates or held higher qualifications, and 27 (18%) were illiterate.

The medical history of the participants revealed that a significant number, 69 individuals (46%) had been living with diabetes for over 10 years. A positive family history of diabetes was reported by 86 participants (57.3%), prior episodes of foot ulcers were noted in 57 participants (38%) and retinopathy, another common diabetes-related complication, was present in 44 participants (29.3%). Regarding current diabetes medication, 74 participants (49.3%) were on oral hypoglycaemics, 43 (28.7%) were on both oral hypoglycaemics and insulin, and 33 (22%) were on insulin only (Table 2).

**Table 2:** Baseline Profile and Medical History of Participants (N=150)

Variable	Categories	Frequency (n)	Percentage (%)
Gender	Female	73	48.7
	Male	77	51.3
Age categories (years)	18–30	16	10.7
	31-45	42	28.0
	46–60	59	39.3
	>60	33	22.0
Residential area	Rural	86	57.3
	Urban	64	42.7
Education level	Illiterate	27	18.0
	Primary	45	30.0
	Secondary	46	30.7
	Graduate or Higher	32	21.3
Duration of diabetes	<5 years	23	15.3
	5-10 years	58	38.7
	>10 years	69	46.0
Family history of diabetes	Yes	86	57.3
	No	64	42.7
History of foot ulcers	Yes	57	38.0
	No	93	62.0
History of retinopathy	Yes	44	29.3
	No	106	70.7
Current diabetes medication	Oral hypoglycaemics	74	49.3
	Insulin	33	22.0
	Both	43	28.7
Smoking history	Yes	65	43.3
	No	85	56.7
Alcohol consumption	Yes	52	34.7
	No	98	65.3
Hypertension	Yes	81	54.0
	No	69	46.0
Peripheral neuropathy	Yes	92	61.3
	No	58	38.7
Peripheral vascular disease	Yes	72	48.0
	No	78	52.0
Duration before medical attention	<1 week	44	29.3
	1-4 weeks	73	48.7
	>4 weeks	33	22.0
Reasons for delayed presentation	Financial constraints	55	36.7
	Lack of awareness	43	28.7
	Fear of amputation	36	24.0
	Distance to healthcare	16	10.6
Compliance to diabetes treatment	Regular	96	64.0
	Irregular	32	21.3
	None	22	14.7
Adherence to lifestyle changes	Excellent	30	20.0
	Good	48	32.0
	Poor	44	29.3
	None	28	18.7

Ulcer characteristics and DUSS component distribution

An analysis of ulcer characteristics revealed that 72 participants (48%) presented with deep ulcers, 63 (42%) had superficial ulcers, and 15 (10%) exhibited ulcers with bone involvement. Regarding infection status, mild infection was prevalent in 77 participants (51.4%) and severe infection in 35 (23.3%). Ischemia was also a notable feature, with mild ischemia present in 63 participants (42%), severe ischemia in 35 (23.3%), and no ischemia in 52 (34.7%) (Table 3).

**Table 3:** Clinical Characteristics of Diabetic Foot Ulcers Among Study Participants (N=150) Note: These characteristics were recorded for descriptive analysis and are not part of the Diabetic Ulcer Severity Score (DUSS) scoring system. DFU: Diabetic foot ulcer.

Clinical Characteristic of DFUs	Categories	Frequency (n)	Percentage (%)
Ulcer depth	Superficial	63	42.0
	Deep	72	48.0
	Bone involvement	15	10.0
Presence of infection	None	38	25.3
	Mild	77	51.4
	Severe	35	23.3
Presence of ischemia	None	52	34.7
	Mild	63	42.0
	Severe	35	23.3

The components of the DUSS were individually assessed. Palpable pedal pulses were present in 105 (70%) participants. Probing to bone was observed in 33 (22%) participants. The ulcer site was predominantly the foot, affecting 115 (77%), while toe ulcers were present in 35 (23%) participants. A single ulcer was noted in 81 (54%), and multiple ulcers were present in 69 (46%) subjects (Table 4).

**Table 4:** Distribution based on components Used for DUSS Score Calculation (N=150) DUSS: Diabetic Ulcer Severity Score

DUSS Component	Categories	Frequency (n)	Percentage (%)
Palpable pedal pulses	Present	105	70.0
	Absent	45	30.0
Probing to bone	No	117	78.0
	Yes	33	22.0
Ulcer site	Toe	35	23.0
	Foot	115	77.0
Number of ulcers	Single	81	54.0
	Multiple	69	46.0

DUSS score distribution and associated management outcomes

The distribution of DUSS scores among the participants showed that the highest frequency was observed at a score of two, accounting for 54 patients (36%). A DUSS score of one was found in 50 patients (33.3%), while 28 patients (18.7%) had a score of three. The lowest scores were observed for DUSS of 0 (13 patients, 8.7%) and DUSS of four (five patients, 3.3%).

The management strategies employed were correlated with the DUSS scores. Amputation (minor and major) was the most common intervention overall, performed in 88 out of 150 patients (59%), followed by surgical debridement in 54 (36%) and skin grafting in eight (5%) patients. A detailed analysis based on DUSS scores highlighted an important clinical pattern: all 28 patients with a score of three and all five patients with a score of four required amputation. In comparison, those with lower DUSS scores received a wider variety of treatment options. However, seven (53.8) patients with a DUSS score of 0 and 13 (26%) patients with a DUSS score of one needed minor amputations as well (Table 5).

**Table 5:** Distribution of DUSS Scores and Associated Management Outcomes (N=150) DUSS: Diabetic Ulcer Severity Score

DUSS Score	Surgical Debridement (n)	Skin Grafting (n)	Minor Amputation (n)	Major Amputation (n)	Total (n)
0	5	1	7	0	13
1	32	4	13	1	50
2	17	3	26	8	54
3	0	0	9	19	28
4	0	0	1	4	5
Total	54	8	56	32	150

Association of DUSS score with length of hospital stay and amputation rates

A significant association was found between DUSS scores and the duration of hospital stay. The Chi-square test produced a value of 64.9 with a p-value of <0.00001, showing that higher DUSS scores were strongly linked to longer hospital stays. Notably, all 13 patients with a DUSS score of zero were hospitalized for up to seven days. Among those with a DUSS score of one, 28 patients (56%) stayed for up to seven days, while 22 patients (44%) remained hospitalized for eight to 30 days. Among patients with a DUSS score of two, eight were hospitalized for up to seven days, 40 (74%) stayed between eight and 30 days, and six (11%) required 31 to 90 days of hospitalization. For those with a DUSS score of three, 19 patients (68%) stayed eight to 30 days, while nine patients (32%) were admitted for 31 to 90 days. All five patients with a DUSS score of four required hospital stays ranging from 31 to 90 days (Table 6).

**Table 6:** Association of DUSS Score with Length of Hospital Stay in days (N=150) DUSS: Diabetic Ulcer Severity Score

DUSS Score	Length of stay in hospital (days)		
	Upto 7 days	8-30 days	31-90 days
0	13	0	0
1	28	22	0
2	8	40	6
3	0	19	9
4	0	0	5
Total	49	81	20
Statistical Significance - χ² = 64.9 p<0.00001			

A statistically significant relationship was also observed between DUSS scores and the need for amputation. The Chi-square test yielded a value of 36.9 with a p-value of <0.00001, indicating that higher DUSS scores were closely linked to a greater risk of amputation. Among patients with a DUSS score of zero, seven out of 13 (53.8%) underwent amputation. For those with a score of one, 14 out of 50 (28%) required amputation. The rate rose sharply with increasing scores: 34 out of 54 patients (62.9%) with a DUSS score of two underwent amputation. Notably, all patients with scores of three (28 patients) and four (5 patients) required amputation (Table 7).

**Table 7:** Association of DUSS Score with rate of amputations (Minor/major) (N=150) DUSS: Diabetic Ulcer Severity Score

DUSS Score	Total patients having the score	Amputation (minor/major), n	Percentage (%)
0	13	7	53.8
1	50	14	28
2	54	34	62.9
3	28	28	100
4	5	5	100
Total	150	88	58.6
Statistical Significance χ² = 36.9 p < 0.00001			

## Discussion

The findings of the study provide compelling evidence regarding the utility of DUSS as a prognostic tool and highlight several critical aspects of DFU management in this region. Diabetic foot is a common and difficult complication of diabetes, with foot infections affecting an estimated 26% to 34% of diabetic patients in India [12]. The study's results, encompassing 150 DFU patients at RIMS, Ranchi, demonstrate a significant association between higher DUSS scores and adverse clinical outcomes, specifically prolonged hospitalization and an increased need for amputation. These observations align with previous research that has utilized DUSS for risk stratification in DFU management [9,13,14].

The study cohort consisted of 51.3% men and 48.7% women, with most participants (39.3%) aged 46-60 years. A large share (57.3%) came from rural areas and nearly half (46%) had lived with diabetes for over 10 years. The high prevalence of long-standing diabetes, peripheral neuropathy (61.3%), and peripheral vascular disease (48%) indicates a high-risk group prone to severe diabetic foot ulcer (DFU) complications. By the time these patients reach a tertiary centre like RIMS, their ulcers are often advanced, as reflected in the high rates of deep ulcers (48%), mild infections (51.4%), and mild ischemia (42%). This context underscores the importance of the DUSS score's predictive power, as it is used in a population already vulnerable to serious outcomes, highlighting its relevance for guiding treatment and resource allocation [15]. 

The study's sample size (N=150) is comparable to other notable DFU studies as well [11,12]. While a study by Edmonds demonstrated improved healing rates with innovative skin grafting techniques, only a small proportion of patients in the current study underwent skin grafting [10]. This disparity suggests a critical gap in the local management of DFUs: by the time patients arrive at RIMS, their ulcers are often too advanced for less invasive reconstructive options, frequently necessitating more drastic interventions like amputation. This points to a systemic issue where preventative care and early intervention at primary or secondary levels may be insufficient, leading to a higher burden on tertiary centres for aggressive procedures. Furthermore, the finding that delays in seeking treatment significantly increased amputation rates (a 59% amputation rate among patients, with 22% presenting after four weeks of ulcer onset) is consistent with observations by Lipsky et al. [15]. This reinforces the importance of patient education and timely access to care in mitigating adverse DFU outcomes. The high prevalence of neuropathy (61.3%) and peripheral vascular disease (48%) among DFU patients in this study, compared to a lower high-risk prevalence (11.3%) in a study by Erdem et al. [16]. This further emphasizes the advanced stage of disease often encountered in this tertiary care setting. 

The assessment of DUSS scores revealed that 69.3% of patients had scores of one or two. Among the 88 patients who ultimately underwent amputation, 62 had DUSS scores of two or three. There was a very strong and statistically significant link between higher DUSS scores and the risk of amputation (p<0.00001). Importantly, every patient with a DUSS score of three or four required amputation. This observation establishes a clear clinical threshold for DUSS, suggesting that these levels of severity often necessitate limb salvage procedures. For lower scores (zero, one, and two), patients received varied treatments, including minor amputation, debridement, or skin grafting, depending on the presentation of the wound. These results align with the findings of studies by Kumar et al., Menezes et al., and George et al., further supporting the DUSS as a reliable tool for predicting clinical outcomes [14,17,18]. The consistent finding across multiple studies that higher DUSS scores correlate with increased amputation rates and prolonged hospital stays elevates DUSS from a mere classification system to a robust, universally applicable prognostic and decision-making tool.

A noteworthy finding from our study was that seven out of 13 cases (53.8%) among patients with a DUSS score of zero had undergone minor amputations, which may seem contradictory to the DUSS’s predictive value. However, several factors may account for this apparent anomaly. Firstly, the DUSS score primarily assesses local ulcer characteristics and does not incorporate systemic clinical parameters such as hemodynamic instability, sepsis, and critical limb ischemia, which may have strongly influenced surgical decision-making. Secondly, the application of the DUSS scoring system at the point of data collection may have resulted in the underestimation of initial ulcer severity, because some patients present to the tertiary care centres after initial treatment elsewhere, which may have led to a discrepancy between the clinical status at presentation and the DUSS score recorded. This finding shows that though the DUSS tool is a validated tool, it may not be completely predictive in real-life clinical settings and there is a need to integrate clinical judgement and broader patient assessment alongside scoring tools for comprehensive decision-making. The revision of diabetic ulcer scoring systems may benefit from including vascular imaging findings, systemic infection markers, and comorbidity indices to enhance their prognostic accuracy in tertiary care settings. However, for primary and secondary level health care systems, DUSS can still be very important for prognosis when patients present at earlier stages of ulcer development.

Surgical debridement was the second most common treatment modality, performed in 54 patients, with 32 of these having a DUSS score of one. It is noteworthy that no patients with DUSS scores of three or four underwent debridement or skin grafting, suggesting that these higher scores indicate tissue damage too severe for conservative treatments. This aligns with Saraswat et al., who reported increased amputation rates and longer recovery times in patients with higher DUSS scores [19]. 

Furthermore, the duration of hospitalization significantly increased with higher DUSS scores (p<0.00001). Patients requiring amputation experienced significantly longer hospital stays compared to those who underwent surgical debridement or skin grafting. This observation is consistent with Beckert et al., who developed the DUSS and demonstrated that each incremental increase in the score corresponded to a 35% reduction in the probability of healing, alongside a higher likelihood of requiring surgical intervention or hospitalization [9]. Similarly, Kim et al. found that factors such as elevated erythrocyte sedimentation rate (ESR), higher glycated haemoglobin (HbA1c) levels, increased body mass index (BMI), and the presence of major vascular diseases contributed to prolonged hospitalization in patients with infected DFUs [20]. Another study by Da Ros et al. also reported that severe DFUs, particularly those complicated by infection and ischemia, significantly prolong hospital stays and elevate treatment costs [21]. The consistent correlation between DUSS and hospitalization duration reinforces the score's utility in predicting healthcare resource utilization. 

The prevalent risk factors identified in this cohort, including smoking (43.3%), alcohol consumption (34.7%), hypertension (54%), peripheral neuropathy (61.3%), and peripheral vascular disease (48%), likely contributed to the observed ulcer severity. The results are supported by Parveen et al. who also noted that comorbid conditions and lifestyle factors exacerbate DFU severity, thereby elevating DUSS and complicating treatment outcomes [22]. 

The study also highlighted the impact of delayed presentation on clinical outcomes. Nearly half of the participants (48.7%) presented between one and four weeks after symptom onset, with significant proportions attributing the delay to financial constraints (36.7%), lack of awareness (28.7%), fear of amputation (24%), and distance to healthcare facilities (10.6%). These factors, which exacerbate DFU severity and complicate management, have been similarly documented in prior DUSS studies [23,24]. The facts, such as patients leaving against medical advice, non-compliance with oral medications, and deaths from metabolic complication, are not merely methodological challenges but are themselves reflections of the complex realities in managing DFU patients in this setting. These real-world barriers to care likely contribute to the observed adverse outcomes and highlight the multifactorial nature of DFU progression beyond the ulcer itself, including patient adherence and the severity of systemic complications. Therefore, there is a need for interventions at primary and secondary level public health facilities to aid in early detection, patient education, and enhanced access to care at primary and secondary healthcare levels to alleviate severe complications.

This study has several limitations that must be acknowledged. First, the DUSS score does not encompass all factors influencing ulcer healing and outcomes, such as duration of diabetes, glycaemic control, delay in presentation, or adherence to medical advice. Therefore, outcomes such as the occurrence of amputations in low DUSS score patients may reflect variables not captured by the scoring tool. Second, the study design and duration restrict our ability to establish causal relationships or assess long-term outcomes. Third, although data collection followed a systematic protocol, the reliance on clinical judgement for parameters like ulcer depth or presence of infection may introduce some degree of inter-observer variability. Finally, the study was conducted in a single tertiary care centre, which may limit the generalizability of the findings to other settings with different resources or patient profiles.

## Conclusions

The study shows that the DUSS scoring system offers useful guidance in stratifying the severity of DFUs and correlates well with the need for more extensive surgical interventions, particularly major amputations, which were largely confined to patients with higher DUSS scores. However, an important finding was that some patients with a DUSS score of zero still required minor amputations, which was because of localized complications not taken by the scoring system. This points towards an important fact that while DUSS is effective in predicting the overall severity of ulcers, it does not account for systemic or vascular factors that may independently influence treatment decisions in clinical settings.

Despite this, the DUSS remains a valuable and practical tool, especially for early risk stratification and guiding timely interventions. Incorporating DUSS into diabetic foot care pathways can enhance clinical decision-making, promote limb preservation, and contribute to more efficient resource utilization, when used in lower strata of healthcare. And, its use would be beneficial alongside broader clinical judgment and assessment tools in tertiary care settings.
